# Progress in Neurology

**Published:** 1902-09-20

**Authors:** 


### Progress in Neurology.

(*Continued from page* 398.)

**Ankle-clonus.**—Weir-Mitchell[5] says that it is commonly thought that in ankle-clonus both the gastrocnemius and soleus respond to the pull made on them by the dorso-flexion of the foot. He has found, however, in a large number of cases that it is only the soleus which responds—the gastrocnemius remaining passive. The proof is easily elicited in thin spastic patients by putting the patient supine with the leg fully flexed on the thigh. As the foot is flexed by the hand it will be found that the gastrocnemius is felt to become tense, but that, as extension occurs, no motion takes place in the belly of that muscle. The active agent in the series of jerks is only the soleus which can be felt to harden with each jerk. The explanation of the fact seems to lie largely in the mechanical relations and attachments of the two muscles, and also in the fact that the gastrocnemius is less excitable than the soleus.

**Laryngeal Crises in Tabes Dorsalis.**—Hughlings Jackson[6] has recently discussed the nature of the laryngeal crises in tabes dorsalis, which he regards s bulbar fits. The convulsions and loss of consciousness which may occur in such fits result from asphyxia set up by spasm of the muscles closing the glottis and perhaps from spasm of other muscles of the thoracic cage. In normal conditions the abductor and adductor muscles of the vocal cords act in amicable antagonism, when the glottis is widened there being a preponderating action of the abductor muscles with moderating action of the adductors, whilst the reverse takes place when the glottis is narrowed. The respiratory centre acts on the vocal cords by intermediation of the subordinate abductor and adductor motor centres. The mechanism of the paroxysm is as follows :—An impulse from the interior of the larynx puts the medulla sensory centres into great activity, and provoke great over-activity of the healthy respiratory centre, from which impulses pass to the motor laryngeal centres. Now the abductor centres are weak, as shown by the investigations of Semon, so that the total result is a narrowing of the glottis. This is the first stage of the crisis. A second often follows—the narrowing of the glottis causes asphyxia, and the supervenous blood stimulates the respiratory centre, so that the asphyxia increases in compound degree. The loss of consciousness may be due to upward discharges from the respiratory centre or to supervenosity ; and the convulsions to the abnormally strong discharges from the respiratory centre, or possibly are directly induced by the supervenous blood acting on the spinal centres.

**Diphtheria.**—Box[7] has recorded a case of ataxy and tetany occurring as sequelæ of a case of diphtheria. Three weeks after the beginning of the disease tetany supervened, marked in both hands and feet ; this gradually improved, though slight spasms occurred in the arms for many weeks. Five weeks subsequently ataxy came on, with weakness and hyperæsthesia of the legs. The patient could not stand, and in attempting to walk reeled in all directions, and the movements of the arms were inco-ordinate and almost choreic. Muscular weakness was inconsiderable, and the ataxic movements were very forcible. This ataxy was probably due to peripheral neuritis, preventing the transmission of afferent impulses, principally from the muscles and bones on which the sense of position depends. Both tetany and ataxy, without muscular impairment, are exceedingly rare as sequelæ of diphtheria. Phrenic paralysis is also a sequela of diphtheria, and two cases have been observed by McKensie.[8] The clinical features were—the absence of abdominal movement, with abdominal carination, monosyllabic, whispering, voice, vomiting at the onset but not afterwards, the development of pneumonia, and, finally, death from heart failure. The mechanism of the condition is that owing to the non-descent of the diaphragm congestion of the bases sets in, and this congestion is succeeded by the development of a septic inflammation.

[5] Jour. of Nerv. and Ment. Disease, May, 1902. [6] Lancet, March 15, 1902. St. Thomas's Hospital Reports, Vol. 28. [8] Intercolonial Med. Jour., Nov. 20, 1901.

